# Hospitals and Unemployment

**Published:** 1922-02

**Authors:** 


### HOSPITALS AND UNEMPLOYMENT.

### THE KING'S FUND AND THE CABINET COMMITTEE.

THE following correspondence has passed between King Edward's Hospital Fund and the Cabinet Committee on Unemployment :—

*To the Rt. Hon. Sir Alfred Mond, Bt., M.P., Chairman, Cabinet Committee on Unemployment.*

SIR,—The Executive Committee of King Edward's Hospital Fund desire to bring to the notice of the Cabinet Committee on Unemployment the fact that at some of the London Hospitals painting, repairs and renewals are long overdue, having been deferred first because of the war and subsequently through shortage of funds. At other hospitals urgently-needed schemes of improvement, also deferred during the war, have been prepared and could and would have been already put in hand were it not for the present financial difficulties of the hospitals.

The plans and specifications are in many cases ready and the work could, therefore, be speedily begun as soon as the necessary funds were available, without the extravagance which is almost inseparable from any large operations undertaken, without the necessary months of consideration and planning. In many cases, also, part of the money is in hand and could be used for the provision of immediate employment if supplemented out of funds intended for the relief of unemployment.

This would be work of public utility to the borough in which the hospital is situated, to London as a whole, and to the country generally.

Moreover, contributions towards this work would not only have the advantage of assisting the unemployed and the sick, but also, by helping the voluntary hospitals to tide over their present difficulties, would be in line with the recommendations of Lord Cave's Committee and would reduce the risk of the hospitals becoming a permanent charge on State or Municipal funds.

If this suggestion meets with approval, the King's Fund would be pleased, if so desired, to communicate with the hospitals of London and to prepare a schedule of the works which the hospitals would wish to submit for consideration, together with such preliminary particulars as the authorities might require. In many cases the plans have already been submitted by the hospitals to the Fund in the ordinary course and passed as urgent, subject to the hospital being able to see its way to the necessary funds.

The Executive Committee desire to publish this letter unless you have any objection to their doing so.

We are, sir, Your obedient servants,

SOMERLEYTON,
(*Signed*) FREDERICK M. FRY,
ALAN C. ANDERSON,
*Honorary Secretaries.*

---

FROM THE MINISTRY OF HEALTH TO THE HONORARY SECRETARIES, KING EDWARD'S HOSPITAL FUND FOR LONDON.

MY LORD AND GENTLEMEN.—I am directed by Sir Alfred Mond to refer to your letter of October 11th in which you bring to his notice the proposal of the King Edward's Hospital Fund for the provision of work for the relief of unemployment by the acceleration of the work of repair and redecoration in London hospitals. The question whether such work would be eligible for financial assistance from the Government is one for the decision of the Unemployment Grants Committee within whose jurisdiction lies the distribution of monies voted by Parliament specifically for the relief of unemployment by the provision of such work, and the Minister therefore suggests that an application should be made to that Committee.

Sir Alfred trusts that on examination in detail of the proposals outlined in your letter the Committee, to whom copies of the correspondence have been forwarded, will find that they are eligible, under the terms of the Vote, for financial assistance.

I am to add with reference to the last paragraph of your letter that Sir Alfred has no objection to the publication of this correspondence.

I am, My Lord and Gentlemen,
Your obedient servant,
(*Signed*) W. ARTHUR ROBINSON.

---

FROM KING EDWARD'S HOSPITAL FUND FOR LONDON.

*December 8th*, 1921.

*The Secretary, Unemployment Grants Committee.*

SIR,—I am directed by the Executive Committee of this fund to forward to you the enclosed copy of correspondence between the fund and Sir Alfred Mond, as Chairman of the Cabinet Committee on Unemployment, and to express the hope that the Unemployment Grants Committee will give favourable consideration to schemes of work referred to in that correspondence.

The Executive Committee would be pleased to take immediate steps to obtain and submit any further particulars which your Committee may require in connection with this application.

I am, sir, Your obedient servant,
(*Signed*) H. R. MAYNARD,
*Secretary.*

---

THE friends of Mr. Richard Kershaw, Secretary of the Central London Throat, Nose and Ear Hospital, will have been delighted at the presentation lately made to him, after forty-three years' service. When handing to Mr. Kershaw a purse containing over two hundred guineas, Sir James Dundas Grant explained that this was given by the general committee, the medical staff and some of the former students. Mr. Kershaw has made the finance of special hospitals his particular study, and the position of his hospital in this respect is one of which he is naturally proud. While we must applaud the generosity of his friends in making this presentation, it none the less draws attention to the need for some such organised pension scheme for hospital managers such as that of Sir James Michelli. The time is going by when long service of this character is acknowledged on haphazard lines, and at the discretion of different hospital committees.

